# What Affects Older Adults’ Viewing Behaviors in Neighborhood Open Space: A Study in Hong Kong

**DOI:** 10.3390/ijerph18052430

**Published:** 2021-03-02

**Authors:** Mu-Fei He, Shu-Lin Shi, Ming-Yi He, Yan-Peng Leng, Shao-Yi Wang

**Affiliations:** Department of Landscape Architecture, School of Architecture, Tsinghua University, Beijing 100084, China; hmf18@mails.tsinghua.edu.cn (M.-F.H.); hemy17@mails.tsinghua.edu.cn (M.-Y.H.); lengyp18@mails.tsinghua.edu.cn (Y.-P.L.); wangshao17@mails.tsinghua.edu.cn (S.-Y.W.)

**Keywords:** older adults, neighborhood open space (NOS), viewing behavior, spatio-temporal analysis, quality of life

## Abstract

Research on older adults’ behaviors, living environments, and their quality of life (QoL) has grown rapidly. Viewing behaviors, although broadly acknowledged as critical for older adults’ QoL, have not been systematically examined in situ. What affects the viewing behaviors of older adults in neighborhood open space (NOS) is still unclear. This study conducted unobtrusive continuous observations in NOS of two residential estates in Hong Kong. With spatio-temporal analyses with ArcGIS Pro and statistical analyses with SPSS, principal influential factors to viewing behaviors of older adults in NOS were identified, including distances for viewing, landscape attractiveness, body supporting elements, as well as moving and interactive behaviors. How these factors would affect older adults’ well-being and QoL is discussed from the perspectives of supportive landscape design, sense of control, prospect and refuge, and social support, etc. Corresponding design implications are proposed to enrich existing NOS design knowledge for older adults’ quality of life.

## 1. Introduction

According to Department of Economic and Social Affairs, UN, it is projected that the proportion of older adults, i.e., people who aged 65 or above, will reach 16% in 2050, from 9% in 2019 [1]. Such a fast population aging progress has received increasing attention globally. One fundamental issue of population aging is how to sustain and improve older adults’ quality of life (QoL), which mainly focuses on health (physically, mentally, and socially) and life satisfaction [2,3].

Despite different cultural backgrounds, most older adults prefer living in communities that they are more familiar with rather than being taken care of in institutions [4,5]. However, due to declined health status, they would be more confined to their immediate residential environments as they age [6,7]. Under such circumstances, neighborhood open space (NOS) would play an important role in sustaining or even improving older adults’ QoL. Thus, NOS has received much academic attention [8]. Some studies focus on physical settings, and pay special attention to spatial configurations and natural elements, while some concern social interactions, behavioral patterns, and other social factors [7,9]. Accumulating evidence reveals that NOS could contribute to QoL of older adults by providing spaces for outdoor activities, natural elements that are beneficial to older adults’ physical and mental well-being, and opportunities for various interactions that would contribute to social well-being [8,10,11].

## 2. Literature Review

### 2.1. NOS and Behavior Patterns of Older Adults

Peer-reviewed journal articles and academic books that related to NOS and behavior patterns of older adults were searched in databases of Web of Science and Google Scholar, with key words of “physical activities”, “active aging”, “viewing”, “natural scene”, “visual behavior”, and “sensory perception”. In these studies, both objective and subjective research methods have been employed to measure the relationships between NOS settings, older adults’ behaviors, and their well-being outcomes. Generally speaking, through the past decades, NOS has been admitted as one type of the most convenient and accessible outdoor spaces for older adults to get in touch with nature and public life on a daily basis [12,13,14].

Many studies investigating the contributions of NOS attributes and characteristics to older adults’ QoL revealed positive results. For instance, accessibility [15], connectivity of pedestrian pathways [16], number of destinations [17,18], safety of the space [13], sizes of space, aesthetic attributes [13,15], and amenities or recreational facilities in an open space [17] have been found to encourage physical activity participation among older adults. Meanwhile, natural elements in NOS, including shadowy trees and ornamental plants, are essential for thermal comfort, besides their great aesthetic values [8,19]. Furthermore, such natural elements would become food sources and habitats or niches for urban wildlife, such as birds, butterflies, and dragonflies, which would greatly increase the liveliness of NOS [20,21]. These are commonly attractive to and liked by older adults [22,23], and could trigger interactions between people and environments [23], hence help to promote the use of NOS and positive well-being feedback [8,19]. Actually, it is evident that the psychological benefits of NOS are to release older adults’ stress and reduce depression [24,25]. From this aspect, NOS offers positive distractions and effective being away opportunities, therefore it is healing for older adults [24,26,27].

As stressed in many studies, the physical settings of NOS would affect older adults’ behavior patterns [28,29]. For older adults, the most commonly observed and reported behaviors in NOS include walking, regular exercising, sitting, chatting, etc. [8,11,12]. Their general well-being benefits have been broadly discussed. It is commonly agreed that the behavior patterns of people would be largely affected by the physical settings of NOS, while mutual adaptions exist in some behaviors [30].

It is noticeable that most studied behaviors of older adults involve large extents of body movements or active engagements. Such energy-consuming behaviors would not last long each day for the majority of older adults, considering their limited stamina [12]. Meanwhile, as older adults commonly have a relatively long leisure time, many of them have problems in having something meaningful to pass each day [31]. A parallel study conducted by the authors indicates that some passive behaviors, such as viewing, are quite common among older adults in NOS [32]. However, not much attention has been paid to older adults’ viewing behaviors in NOS, which may be beneficial to QoL.

### 2.2. General Significance of Viewing Behavior

Among all the senses of human beings, vision is probably the most important one for normal people, as “far more of the primate brain is engaged in processing visual information than in processing information from any of the other senses” [33,34]. One may not realize how much he/she relies on vision until it is defunctionalized or constrained due to illness, accident, darkness, etc. Normally, one always wants to have something to settle or rest his/her eyes on while awake, otherwise one may get upset [33]. Compared to other age groups, older adults are probably more in need of this, as they usually have much more leisure time together with less energy, as mentioned above [31]. In such cases, staying in outdoor spaces with natural elements would benefit older adults more, compared to staying indoors [35].

Regarding the benefits of visual behaviors, they could facilitate silent communication in social interactions, and have been proved to significantly influence emotional responses and cognitive attribution in providing information in interpersonal activities [36]. By suggesting a willingness and disinclination of a person’s social attitude [37], visual contacts could help trigger positive interactions in NOS. In fact, simply observing others can be beneficial even without actual intrigued interaction [38,39]. It can not only improve older adults’ mood and self-esteem [40], but also strengthen the sense of connecting with the world beyond one’s body [41,42].

### 2.3. Viewing Behavior of Older Adults in NOS

In the authors’ parallel study mentioned above, a random questionnaire survey on older adults living in public rental housing (PRH) estates in Hong Kong SAR (Hong Kong here after) revealed that although only 6.6% of the participants chose viewing (others’ activities) as one motivation of NOS visits, 56.1% of all participants stated “watching others’ activities” and 49.1% “watching plants or animals” as their actual activities in NOS [32]. These two kinds of viewing behaviors ranked as fourth and sixth by frequency among all of the 23 specified actual activities in NOS.

This not only indicates the potential importance of viewing behaviors, but also their unconscious nature among various behaviors carried out by older adults in NOS. So far, studies in this area generally pay more attention to the objects being viewed and their influence on older adults [43,44,45]. Most of them highly control the views of older adults, including involving selected scenic pictures or certain videos, carefully defining durations of viewing, and pre-constructing feedback, etc. [46,47,48]. However, in reality, older adults in NOS are autonomous on what and how long they look. Additionally, the environments would probably not be defined accurately in the abovementioned studies. Under such circumstances, older adults’ viewing behaviors would be more affected by their physical settings and things happening in the surroundings [49]. Therefore, with the knowledge generated by previous studies on objects of viewing, systematic studies on actual viewing behaviors of older adults should be conducted in situ, so as to understand how viewing behaviors are affected by physical settings, other users, and their activities. Such an understanding would further support NOS designs by bridging knowledge and practice, concerning the actual preference and needs of older adults together with their QoL. Hence, this study was initiated to identify and depict the principal influencing factors of the viewing behaviors of older adults in NOS.

## 3. Methods

### 3.1. Observation Design

#### 3.1.1. Site Background

The investigation was conducted in Hong Kong, taking the same 17 PRH Estates as a parallel study that has been published [32]. On the one hand, the city is facing serious population aging problems, as the number of people aged 65 or above is estimated to double in the next 20 year, with an increase of the population proportion from 18.4% in 2019 to 33.3% in 2039. By 2069, the proportion is projected to reach 38.4% [50]. On the other hand, in compacted Hong Kong, PRH estates are the only residential developments that consistently provide relatively sufficient NOS within each estate, and at the same time are a major provider of affordable accommodations for older adults.

In each of the 17 PRH estates, two to four specific sites were selected from NOS for observation. Each site has a relatively clear boundary, such as enclosing structures, flower beds, pathways, or level differences. Each site should contain covered benches and rubbish bins. All sites in each estate were observed for one weekday (WD), and one weekend or public holiday (WE/PH), applying the standardized record sheet and requirements. All field investigators were trained by the principal investigator beforehand.

Well-being-related information of older adults in these 17 PRH estates was collected in the parallel study mentioned above, with a randomized questionnaire survey (*n* = 426). For smoking, although some older adults did smoke (13.8%), they did not do that in the selected sites as there was no smoking area inside (otherwise the penalty would be HK$1500). Regarding physical and mental health, 41.5% of the participants claimed that they did not have any chronic disease, almost 60% thought their health conditions were good or very good, and only 7% reported poor or very poor health conditions. The most mentioned chronic diseases included cardiovascular and cerebrovascular ones (39.9%), problems related to bones (36.1%), and diabetes (9.6%) [32]. In total, 78% of the participants were satisfied or very satisfied, while 2.4% were dissatisfied or very dissatisfied with their social networks. Generally speaking, older adults in these PRH estates were relatively healthy and could use the NOS freely.

#### 3.1.2. Site Selection

**Figure 1:**
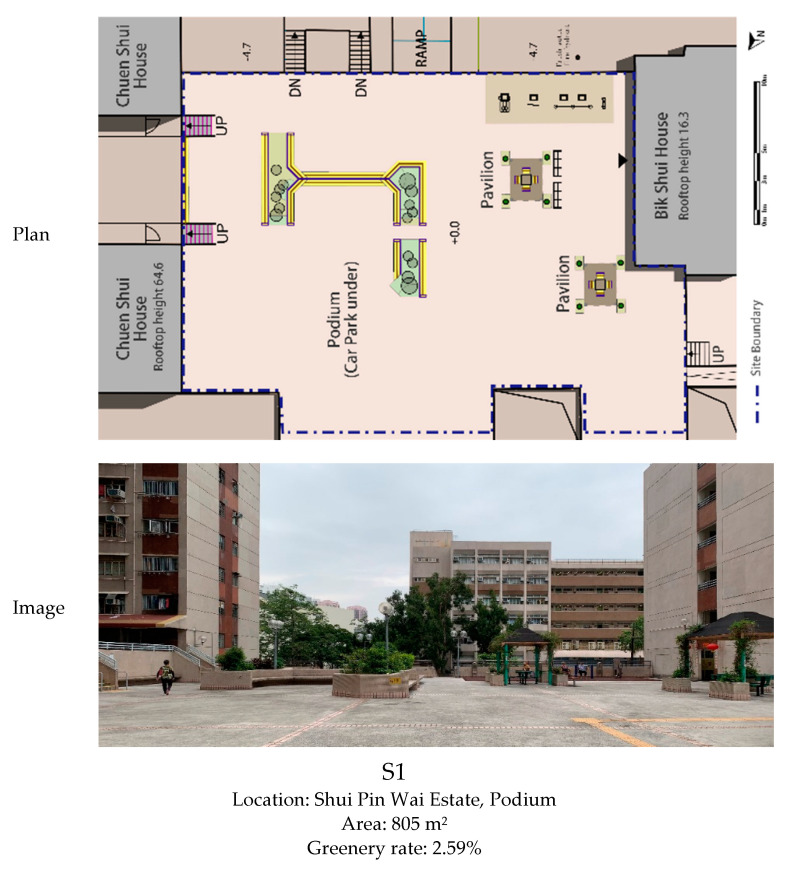

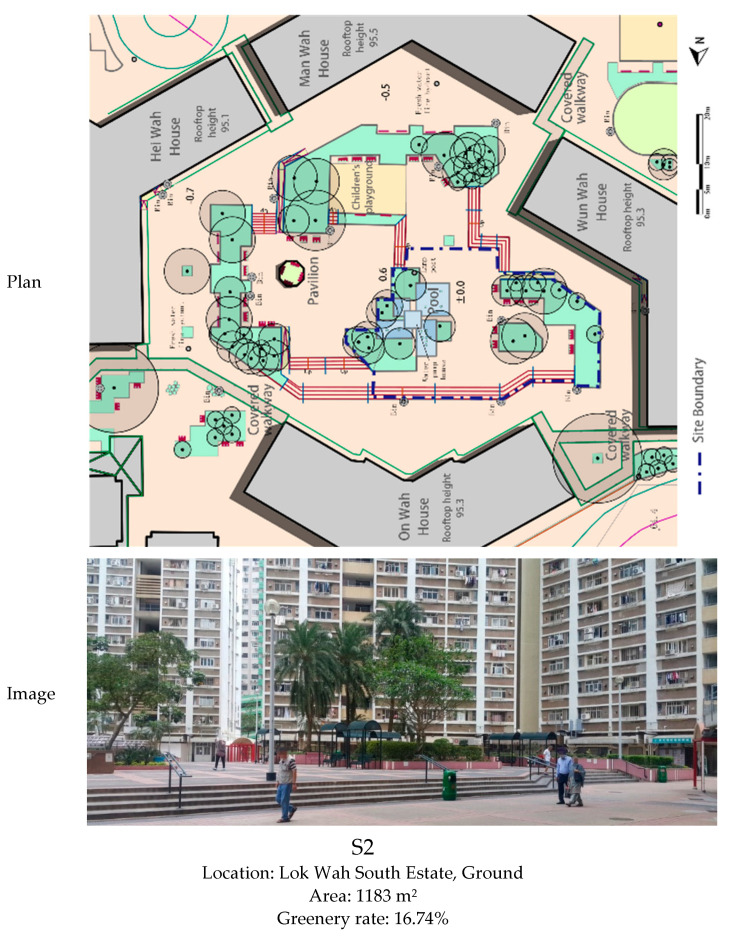
Plans and basic information of the two observed sites.

This study was based on 2 of the 17 PRH estates, both established in the early 1980s and with a high proportion of aged residents (aged 65 or above) by the time of study, i.e., Shui Pin Wai Estate (established in 1981, with 2691 aged residents: 6725 was the total number of residents, i.e., proportion of aged residents: 40.0% [51]) and Lok Wah South Estate (established in 1982, with 4452 aged residents: 12,843 was the total number of residents, i.e., proportion of aged residents: 34.7% [51]). As in PRH estates in Hong Kong, podium NOS usually has a much lower greenery rate and few big trees due to loading constraints; one podium site was selected from Shui Pin Wai Estate (S1), and one ground-level site was selected from Lok Wah South Estate (S2) to cover more types of spatial settings. Other than this, both sites were located in the central area of estates. The plans and basic information of the two sites are shown in Figure 1.

#### 3.1.3. Data Collection and Processing

Unobtrusive continuous on-site observations during 9:00–12:30 and 13:30–18:00, were carried out for S1 on 28 April (WE/PH) and 21 May (WD) 2019, when temperatures were between 23 and 27 °C (73–81 °F); and S2 on 14 May (WD) and 7 June (WE/PH) 2019, when temperatures were between 25 and 33 °C (77–91 °F). Weather on all of the four observation days was cloudy with occasional sunshine.

On each observation day, one investigator was assigned to one site, and stayed in a corner or along the boundaries to minimize their impacts on users and their behaviors. Site users were categorized into pass-by and stay ones. For each pass-by user, the demographic features of gender, estimated age group, walking capacity, whether with company and relationship with company, as well as the route taken and site entering time were marked down on record sheets. The duration of each pass by was standardized as one minute based on the authors’ pilot studies. For each stay user, demographic features and route(s) taken were recorded in the same way as pass-by users. Besides, the starting and ending time, as well as the location for each type of behavior during his/her stay within the site were also recorded besides the overall site entering and exiting time. In addition to paper recording, investigators were also required to take photos of each type of users and behaviors. When there was too much to record instantly, photos were taken for record sorting afterwards.

After on-site observations and record sorting, observation data were input into ArcGIS Pro 2.6 for spatio-temporal analyses. Information in attribute tables was exported for statistical analyses in IBM SPSS 25. Representative on-site photos were also selected for demonstration. Intra- and inter-site comparisons were also conducted on site settings, and the viewing and interactive behaviors of older adults.

## 4. Results

As stated above, OA’s viewing behaviors may be affected by other users and their activities. This study paid special attention to moving and interactive behaviors as they were more dynamic and common according to the observations. The total number of observed users, older adult users (OA here after), non-older adult users (NOA), various stay and OAs’ viewing behaviors, pass-by cases for different groups of users, and all moving and interactive cases on each day and site are summarized below in Table 1.

**Figure 2:**
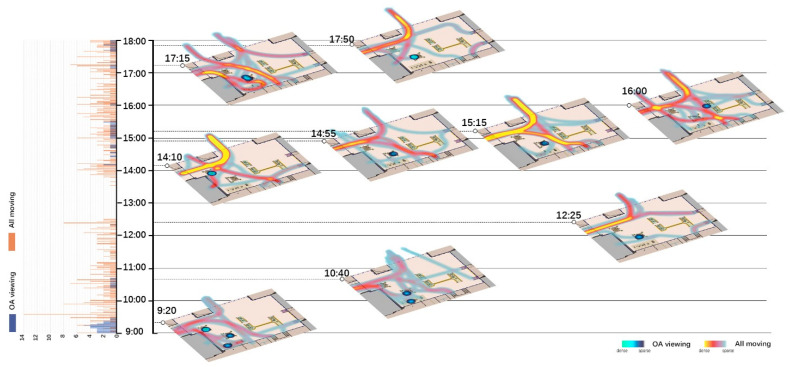
Spatio-temporal relationship between OA-viewing and All-moving behaviors (S1, WD).

**Figure 3:**
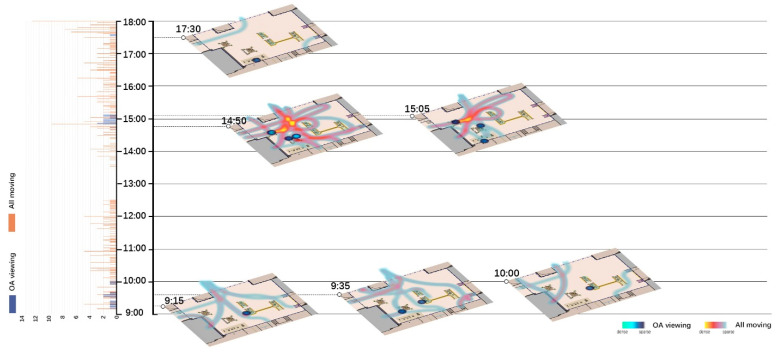
Spatio-temporal relationship between OA-viewing and All-moving behaviors (S1, WE/PH).

**Figure 4:**
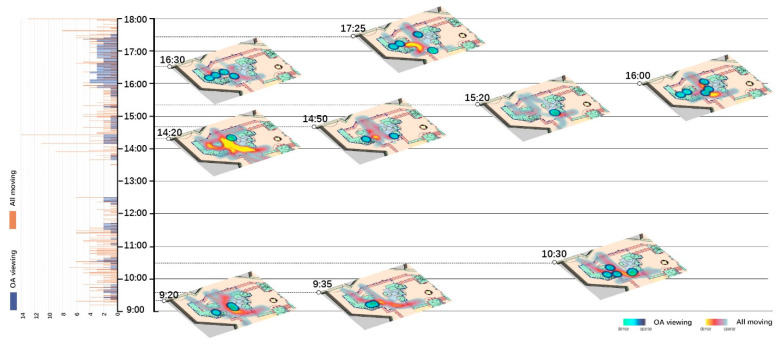
Spatio-temporal relationship between OA-viewing and All-moving behaviors (S2, WD).

**Figure 5:**
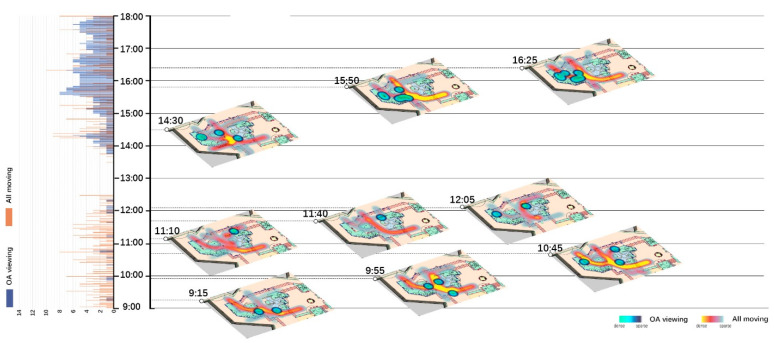
Spatio-temporal relationship between OA-viewing and All-moving behaviors (S2, WE/PH).

Spatio-temporal analyses for all moving behaviors and their relationships with OA-viewing were done in two parts. One is using histograms to show the total number of OA-viewing and total number of All-moving behaviors at each moment, as shown on the left of Figure 2, Figure 3, Figure 4 and Figure 5. The other is visualizing the spatio-temporal distributions of OA-viewing and All-moving behaviors using kernel density estimation (KDE) in ArcGIS Pro. The time slider was set with a 10-min span and 5-min interval, resulting in 96 captures for each site and day. Afterwards, the captured slides were carefully studied integrally with corresponding histograms. Only those that fell into the timeslots with OA-viewing behaviors were further considered. Within this collection of slides, fluctuations in the histograms together with the spatial distributions of the OA-viewing and All-moving behaviors were examined integrally. The final selected slides demonstrated representative and special (1) relationships between OA-viewing and All-moving behaviors, or (2) spatial distributions of OA-viewing behaviors. These final extracted slides of each site and day were then allocated into one graph according to a timeline, as shown on the right of Figure 2, Figure 3, Figure 4 and Figure 5 (OA-viewing as points while All-moving as lines).

**Figure 6:**
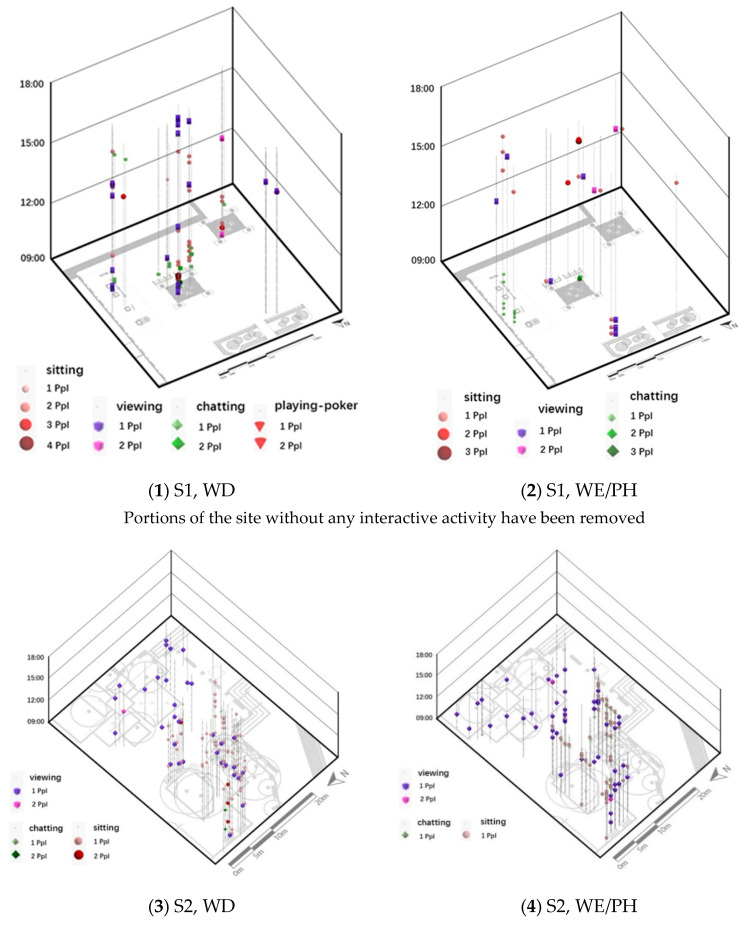
STKDE on OA-viewing and significant OA-interactive and sitting behaviors.

Regarding the interactive behaviors among all users, chatting (*n* = 138), playing poker (*n* = 15), and greeting (*n* = 2) were most frequent during our observations. Due to the different sample sets of the user groups, correlations were only done within the OA user group. As a result, OA-viewing behaviors were found to be significantly correlated with the OA-interactive behaviors of chatting (*n* = 94), while not with playing poker (*n* = 13) or greeting (*n* = 2) (Table 2). The corresponding spatio-temporal kernel density estimation (STKDE) was examined in ArcGIS Pro, as shown in Figure 6 (kernel density represents the number of people (Ppl)). STKDE was chosen for the analysis of interactive behaviors mainly for its advantages in showing highly spatially attached information in point form. The OA-sitting behaviors are also included in Figure 6 as they were found to be highly correlated with OA-viewing (Table 2).

The durations of OA’s stay behaviors are summarized in relation with viewing behaviors in Table 3. Besides all viewing cases, those with sitting or not were summarized separately as sitting was suspected as a critical support for OA-viewing; viewing with other behaviors was summarized to see potential syntheses among OA.

## 5. Discussion

According to the histograms in Figure 2, Figure 3, Figure 4 and Figure 5, clear patterns of OA-viewing behaviors can be identified in both sites. For S1, OA-viewings mainly exist during early mornings, and early and late afternoons, with similar magnitudes; for S2, OA-viewings intermittently occur throughout mornings, and consistently exist with much higher magnitudes during entire afternoons. Such regular patterns lay a good foundation for further discussions.

### 5.1. Spatial Elements and Landscape Factors

Based on the results of the observations, this study implies that the physical attributes of NOS, including spatial elements and landscape characteristics, have a great influence on OA-viewing behaviors in NOS [52]. Such influences are found through examining the distances for viewing, landscape attractiveness, and body supporting elements of the two sites.

#### 5.1.1. Distances for Viewing

An NOS that is supportive of viewing behaviors would probably consist of a good spectrum of distances for viewing. Considering the scope of common PRH estates in Hong Kong, distances for viewing would seldom exceed 70–100 m, which is considered as the cap to clearly see or even recognize others’ behaviors in open outdoor spaces [53]. Given that people can see clearly what they want to see in NOS, distances for viewing would heavily affect viewers’ aesthetic preference, which highly relies on the specific features of targets of interests [44].

Following this logic, S2 provides a wider spectrum of viewing distances for its spatial configuration is relatively more complexed than S1 (Figure 1). This implies that S2 would be more attractive in terms of distances for viewing. Based on Table 1, it can be calculated that for both days, 35.5% among all observed older adults in S1 have viewing behaviors, while the proportion in S2 is 51.8%. According to Table 3, durations of OA-viewings in S2 are almost doubled on WD and more than 2 times longer on WE/PH than S1. These are consistent with findings of previous experts in urban studies. Although hard to specify ranges of distances solely based on this single study, it would be effective to encourage OA-viewing behaviors by providing relatively diverse distances for viewing, i.e., organizing larger and smaller spaces with various proportions flexibly into NOS.

#### 5.1.2. Landscape Attractiveness and Body Supporting Elements

Landscape elements, such as ornamental plants, sculptures, and fountains, are important features in NOS [54]. Through the provision of various sensory stimulations, these elements would allow people to rest their eyes and minds, alleviating fear and mental fatigue, and bringing pleasant psychological feelings [8,55]. According to previous research, plants and water are the two categories of natural elements that are commonly preferred by most people and would lead to the most significant healing effects [40,42]. As shown in Figure 1, in terms of landscape attractions, there are two pavilions with climbers and a set of raised flower beds with flowering shrubs in S1. The site is closely surrounded by residential buildings. In S2, there are fish pools and fountains, sheltered benches that are surrounded by palm trees, and shrubs planted in raised flower beds. Furthermore, adjacent to S2, there are covered walkways as the major pedestrian routes of the estate, a children’s playground, and a large pavilion, which is good for gathering. If only the amount and diversity of attractions are considered, S2 exceeds S1 greatly.

Figure 2, Figure 3, Figure 4, Figure 5 and Figure 6 visualize a striking aggregation around the seating facilities and major attractions among OA-viewings, especially in S2. When examined closely, most benches are arranged in a way that faces the major attractions of fish pools and the adjacent covered walkway in S2 (Figure 1), which allows older adults to enjoy viewing comfortably. Although there is no seating right beside, the fish and turtles inside fish pools make it attractive enough for viewing. In S1, the flower bed area consistently has less OA-viewings compared to the pavilion-fitness area. This is mainly because the combination of the flower bed and benches along its sides (Figure 1) makes flowering shrubs invisible for people sitting on the benches. If the positive effects of sitting on OA-viewing behaviors (rows of viewing—non-sitting and viewing—sitting in Table 3) are considered, the spatial relationships between body supporting elements and attractions appear more influential than simply providing body supporting facilities. Our findings indicate that combinations of body supporting elements and attractions that allow convenient viewings together with strong landscape attractions would significantly increase OA-viewing behaviors in NOS.

### 5.2. Influence of Moving Behaviors

Moving behaviors were the most frequent and dynamic during the observations (Table 1). As people are usually more easily attracted by dynamic objects [54], such moving behaviors are suspected to be influential on OA-viewing behaviors. A positive association (*r* = 0.140, *p* = 0.001) between the number of people moving and that of OA-viewing in each minute was detected with all of the observed data.

As demonstrated in Figure 2, Figure 3, Figure 4 and Figure 5, OA-viewing behaviors tend to appear close to the major routes of moving behaviors while at the same time avoiding stepping on the routes or directly facing pedestrian flows. In this way, older adults would be able to see moving behaviors for a relatively longer time. In other words, the amount of stimulation from each moving object is kind of maximized. Such a prolonged and ever-changing viewing experience may effectively raise interest, reduce boredom, and maintain a certain level of arousal for older adults. Meanwhile, older adults may also feel connected to society, even passively. These are all critical for older adults’ well-being [42,56].

Besides, maintaining visual connections with pedestrian flows may also bring older adults a sense of safety, for they know that timely help could be received if in need [57]. Another possible reason may be rooted in prospect and refuge theory, as people prefer being able to see but not to be seen [58,59]. As shown in Figure 6(3,4), OA-viewing behaviors were mainly distributed around the fish pools and bench area in S2. In both days, the mean duration of observed OA-viewings was 3′51″ in the fish pool areas (*n* = 32, SD = 3′41″), much shorter than 25′12″ in the bench area (*n* = 50, SD = 30′50″). Despite the different seating facility provisions, complete exposure to the surroundings in the fish pool area would be a major reason for such short OA-viewing durations. On the other hand, for S1, with similar prospect and refuge conditions, similar durations of OA-viewings were recorded in the flower bed bench area (mean = 7′12″, *n* = 5, SD = 4′52″) and pavilion-fitness area (mean = 7′15″, *n* = 28, SD = 8′24″). Besides physical settings, the flow of pedestrians would also affect the sense of prospect and refuge. As shown in Figure 2; Figure 3, when the pavilions are surrounded by pedestrian flows, they hardly support OA-viewings. This would not be the case in S2 as all benches are allocated against wide flower beds, which block pedestrians. This may explain the much shorter durations in S1 than those in the bench area in S2.

Viewing people moving around in NOS would be a highly cost-effective way for older adults to keep themselves connected with society and their peers. It also ensures a high sense of control for older adults to decide whether to step forward for more interactions with others or stay comfortably in their little worlds. This is also contributive to older adults’ well-being and QoL [39,60].

### 5.3. Influence of Interactive Behaviors

According to Table 2, OA-viewings were negatively associated with chatting in both sites. Referring to Figure 6, OA-viewing and chatting behaviors were basically distributed apart spatially and temporally, especially for S1-WE/PH, S2-WD. For S1-WD and S2-WE/PH, some OA-viewings were found to co-exist with chatting. However, it is rare to see a label of chatting right above or below a label of viewing along the same spatial indication line. Besides, for S2, no chatting was identified among OA-viewers around the fish pools area in both days. This infers that OA-viewers are more likely to keep viewing as an individual activity rather than mixing up with interactive ones, either intentionally or unconsciously. However, it does not imply that these older adults isolate themselves from or lose interest in their surroundings, as some OA-viewers did sit beside people chatting (S1-WD and S2-WE/PH in Figure 6). In such situations, they may be listening to others and have active minds. This, especially the latter, is especially critical to prevent brain degradation and dementia among older adults [36].

**Figure 7:**
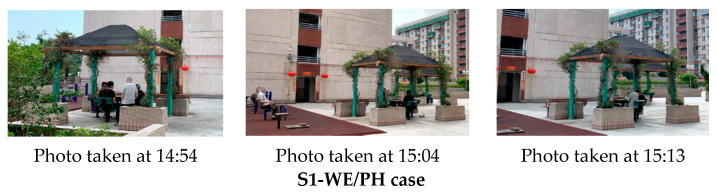

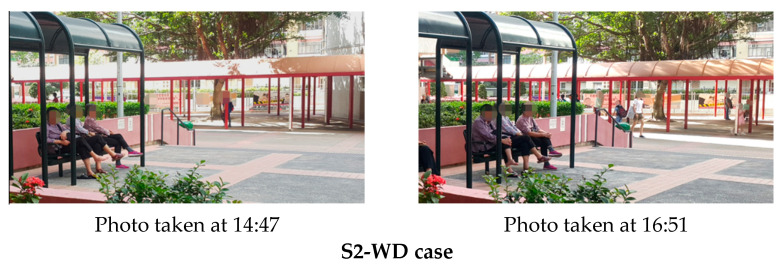
Typical multi-behavioral viewings among OA.

Although no significant association was detected between OA-viewing and greeting or playing poker (Table 2), co-existences and shifts between these activities for individual older adults were observed on site (Figure 6). For example, one older man (in light grey) in S1-WE/PH was observed watching poker playing (viewing) from 14:50 to 15:10, either next to the table or from fitness equipment nearby; starting from 15:11, he joined the poker play until 15:41 (Figure 7). Another case in S2-WD recorded three old ladies who sat on a bench close to one of the site entrances from 14:26 until 17:04. During almost the entire afternoon, they spent most of the time looking at the surrounding people and landscape while talking to each other occasionally (Figure 7). Generally speaking, such a mixture of the mode of viewing with low-intensity interactive activities would effectively enrich the experience and lengthen older adults’ stays in NOS (Table 3).

### 5.4. Design Implication

Based on this study, some design implications emerge for NOS in paying special attention to older adults’ QoL, from the perspective of facilitating viewing behaviors among older adults:

Firstly, it is critical to create multi-distances and angles for viewing landscape attractions within a single space, so as to enrich the viewing experience. This could be achieved by diversifying spatial configurations, together with making good use of ornamental vegetation and allocating them within comfortable view-sheds of people staying in the space.

Secondly, in order to allow older adults to stay comfortably in NOS, properly designed and allocated body supporting facilities should be provided. Such facilities are not limited to benches but could be combined with other landscape elements like edges of flower beds as well. To maximize their contributions, it is better to allocate these facilities along and facing major pedestrian flows, or towards landscape attractions, with good prospect and refuge relationships, besides fulfilling barrier-free and safety requirements. In addition, weather protection, such as shelters, would encourage older adults’ usage.

Thirdly, older adults in NOS usually take viewing as an individual activity without much interaction with others, although they need such social supports from time to time. This suggests NOS designs should provide sufficient space and facilities that are suitable for activities with a relatively lower intensity and small scale, given that quiet viewing is compatible. Meanwhile, considering the public nature of NOS, it would better be able to fulfill different needs of different users as well.

### 5.5. Limitation

As this study focused on the viewing behaviors of older adults in NOS based on two sites on one weekday and one weekend/public holiday each, it would be strengthened if data from more cases could be analyzed together or with a longer period of observations so as to enlarge the sample sizes. In this way, more comprehensive patterns of OA-viewing behaviors and corresponding influencing factors would be revealed with rigorous statistical analyses. Besides, both sites of this study are located in PRH estates, which accommodate lower-income residents of Hong Kong. This may limit behavioral patterns. Therefore, it would be valuable to study NOS that caters various social classes in case social status affects OA-viewing behavior. Thirdly, as unobtrusive observation was employed to avoid disturbing original/actual use patterns, observed users were not asked their reasons for certain behaviors during this study. Although a questionnaire survey on older residents in the same PRH estates was conducted parallel to this observation, it may not directly explain the reasons behind the observed phenomena. Future research could try to fine tune the research method so as to obtain strictly paired objective and subjective data for more convincing interpretations.

## 6. Conclusions

This is probably the first study that investigated the viewing behaviors of older adults in NOS specifically with objective spatio-temporal pattern analyses that were paired with statistical analyses and supported by the results of a parallel subjective questionnaire survey.

The results revealed how OA-viewing behaviors in NOS are affected by spatial configurations and landscape features, as well as by other users and behaviors. Specifically, distances for viewing, landscape attractiveness, and body supporting elements, as well as moving and interactive behaviors were identified as principal influential factors of viewing behaviors among older adults in NOS. How these factors would further affect older adults’ well-being and QoL was discussed from the perspectives of supportive landscape design, sense of control, prospect and refuge, social support, etc. Accordingly, design implications were proposed in terms of spatial configurations, provision of body supporting facilities, as well as the compatibility and flexibility of NOS functions. This enriches current NOS design knowledge for older adults’ QoL and preliminarily confirms a direction for further research.

## Figures and Tables

**Table 1:** Total number of users observed on sites (by category).

Category	Sub-Category	S1-WD	S1-WE	S2-WD	S2-WE
Total no. of users	All	558	284	475	581
	OA	202	108	187	174
	NOA	356	176	288	407
Stay	All	159	134	209	191
	OA	102	74	98	100
	NOA	57	60	111	91
	OA-viewing	22	11	34	52
Pass by	All	399	150	266	390
	OA	150	67	98	97
	NOA	249	83	168	293
Moving *	All	717	418	684	772
Interactive behaviors	All	42	43	29	51

* Moving covers all pass-by behaviors and stay users’ movements from one spot to another during stays. The number of moving cases is larger than the total number of users because many users changed spots during stays, which resulted in extra moving counts.

**Table 2:** Correlation between OA-viewing and sitting and OA-interactive and sitting behaviors.

Site-Date	Indicator	Chatting	Playing Poker	Greeting	Sitting
S1-WD	*r*	−0.243 *	−0.173	−	0.158
	*p*	0.015	0.082	−	0.112
S1-WE/PH	*r*	−0.263 *	−0.086	−0.049	0.279 *
	*p*	0.712	0.463	0.676	0.017
S2-WD	*r*	−0.277 **	−	−0.074	−0.346 **
	*p*	0.006	−	0.466	0.001
S2-WE/PH	*r*	−0.691 **	−	−	−0.259 **
	*p*	0.000	−	−	0.010

* *p* < 0.05 (2-tailed), ** *p* < 0.01 (2-tailed), − sample insufficient.

**Table 3:** Duration of OA’s stay behaviors.

Behavior Category	Indicators	S1-WD	S1-WE/PH	S2-WD	S2-WE/PH
All—viewing	N	22	11	34	52
	Mean	7′44″	6′16″	12′46″	18′38″
	SD	9′10″	5′37′	24′01″	27′11″
Viewing—Non-sitting	N	9	4	17	18
	Mean	3′40″	2′00″	5′42″	2′20″
	SD	3′56″	48″	4′51″	48″
Viewing—Sitting	N	13	7	17	34
	Mean	10′32″	8′42″	19′49″	27′15″
	SD	10′45″	5′45″	32′33″	30′20″
Viewing only	N	0	0	8	2
	Mean	−	−	17′00″	1′00″
	SD	−	−	40′02″	0′00″
Viewing plus other behavior	N	22	11	26	50
	Mean	7′44″	6′16″	11′28″	19′20″
	SD	9′10″	5′37″	17′29″	27′29″

− Not applicable.

## Data Availability

The data presented in this study are available in What affects older adults’ viewing behavior in neighborhood open space: A study in Hong Kong.
